# Myosin heavy chain and cardiac troponin T damage is associated with impaired myofibrillar ATPase activity contributing to sarcomeric dysfunction in Ca^2+^-paradox rat hearts

**DOI:** 10.1007/s11010-017-2954-8

**Published:** 2017-02-17

**Authors:** Árpád Kovács, Judit Kalász, Enikő T. Pásztor, Attila Tóth, Zoltán Papp, Naranjan S. Dhalla, Judit Barta

**Affiliations:** 1 Division of Clinical Physiology, Institute of Cardiology, Faculty of Medicine, University of Debrecen, Debrecen, 4032 Hungary; 2 Research Centre for Molecular Medicine, Faculty of Medicine, University of Debrecen, Debrecen, 4032 Hungary; 3 Department of Physiology and Pathophysiology, Faculty of Health Sciences, St. Boniface Hospital Albrechtsen Research Centre, Institute of Cardiovascular Sciences, College of Medicine, University of Manitoba, 351 Tache Avenue, Winnipeg, MB R2H 2A6 Canada; 4 Department of Cardiology, Institute of Cardiology, Faculty of Medicine, University of Debrecen, 22 Móricz Zs krt., Debrecen, 4032 Hungary

**Keywords:** Calcium paradox, Myofibrillar ATPase activity, Isolated cardiomyocytes, Myofilament protein degradation

## Abstract

This study aimed to explore the potential contribution of myofibrils to contractile dysfunction in Ca^2+^-paradox hearts. Isolated rat hearts were perfused with Krebs–Henseleit solution (Control), followed by Ca^2+^-depletion, and then Ca^2+^-repletion after Ca^2+^-depletion (Ca^2+^-paradox) by Langendorff method. During heart perfusion left ventricular developed pressure (LVDP), end-diastolic pressure (LVEDP), rate of pressure development (+ dP/dt), and pressure decay (-dP/dt) were registered. Control LVDP (127.4 ± 6.1 mmHg) was reduced during Ca^2+^-depletion (9.8 ± 1.3 mmHg) and Ca^2+^-paradox (12.9 ± 1.3 mmHg) with similar decline in +dP/dt and –dP/dt. LVEDP was increased in both Ca^2+^-depletion and Ca^2+^-paradox. Compared to Control, myofibrillar Ca^2+^-stimulated ATPase activity was decreased in the Ca^2+^-depletion group (12.08 ± 0.57 vs. 8.13 ± 0.19 µmol P_i_/mg protein/h), besides unvarying Mg^2+^ ATPase activity, while upon Ca^2+^-paradox myofibrillar Ca^2+^-stimulated ATPase activity was decreased (12.08 ± 0.57 vs. 8.40 ± 0.22 µmol P_i_/mg protein/h), but Mg^2+^ ATPase activity was increased (3.20 ± 0.25 vs. 7.21 ± 0.36 µmol P_i_/mg protein/h). In force measurements of isolated cardiomyocytes at saturating [Ca^2+^], Ca^2+^-depleted cells had lower rate constant of force redevelopment (*k*_tr,max_, 3.85 ± 0.21) and unchanged active tension, while those in Ca^2+^-paradox produced lower active tension (12.12 ± 3.19 kN/m^2^) and *k*_tr,max_ (3.21 ± 23) than cells of Control group (25.07 ± 3.51 and 4.61 ± 22 kN/m^2^, respectively). In biochemical assays, α-myosin heavy chain and cardiac troponin T presented progressive degradation during Ca^2+^-depletion and Ca^2+^-paradox. Our results suggest that contractile impairment in Ca^2+^-paradox partially resides in deranged sarcomeric function and compromised myofibrillar ATPase activity as a result of myofilament protein degradation, such as α-myosin heavy chain and cardiac troponin T. Impaired relaxation seen in Ca^2+^-paradoxical hearts is apparently not related to titin, rather explained by the altered myofibrillar ATPase activity.

## Introduction

Paradoxically, when a physiologically perfused isolated heart is perfused for a short period of time with a Ca^2+^ free, otherwise normal Krebs–Henseleit buffer, and then with buffer ensuring again physiological [Ca^2+^], the heart rapidly deteriorates. This memorable, but unexpected observation was first reported by Zimmerman and Hülsmann five decades ago [1]. Thereafter, this adverse effect of Ca^2+^-repletion on a once Ca^2+^-depleted heart has been known as Ca^2+^-paradox [2]. Although in vivo pathophysiology does not present exclusive depletion and repletion of Ca^2+^ during ischemia and reperfusion (I/R), extreme rise of cytosolic [Ca^2+^] is a key element of I/R injury [3]. For that very reason, an isolated heart undergoing Ca^2+^-paradox has been considered to serve a widely accepted model for investigating the mechanisms of structural and functional myocardial injury due to intracellular Ca^2+^-overload as part of I/R injury [4]. The pathomechanism of Ca^2+^-paradox has been partly explored. The consequence of Ca^2+^-repletion after Ca^2+^-depletion on global left ventricular function is a marked decrease in systolic pressure paralleled by a significant increase in end-diastolic pressure [5, 6]. The rationale of dramatic contractile impairment due to Ca^2+^-paradox is myocardial hypercontracture associated with intense ultrastructural damage [7]. Accordingly, Ca^2+^-depletion initiates a moderate disruption of the adjacent cardiac cells at the intercalated discs [8], β-dystroglycan is dissipated, and cell-to-cell and cell-extracellular matrix connections break up [9]. Finally, in massive Ca^2+^-overload hypercontracture develops [10], while the heart becomes pale because of myoglobin loss [11]. Since cell-to-cell detachment is a necessary step in this phenomenon, isolated cardiomyocytes are thought to avert Ca^2+^-paradox [12, 13]. These observations altogether suggest that contracture is the main cause of contractile dysfunction in Ca^2+^-paradox. In contrast, numerous concomitant intracellular changes have been also described in Ca^2+^-paradox-affected isolated hearts. Ca^2+^-repletion results in excessive Ca^2+^ entry with reverse mode of Na^+^/Ca^2+^ exchanger and transient receptor potential channels [14], as well as through non-specific transmembrane influx [9], sarcolemmal disruption [15] with insufficient sarcolemmal Ca^2+^-activated and Mg^2+^ ATPase unable to extrude Ca^2+^ [16], injured microsomal fraction attended with depressed Ca^2+^-activated and Mg^2+^ ATPase activity, and Ca^2+^ binding of the sarcoplasmic reticulum [17], collectively provoking intracellular Ca^2+^-overload, therefore leading to extensive perturbations of intracellular Ca^2+^ handling. However, it has not been elucidated yet whether sarcomere disruption and myofilament damage also contribute to the contractile failure in Ca^2+^-paradox. In rat models of Ca^2+^-paradox, myosin light chain-1 release [18] and troponin I release together with α-fodrin degradation [19] have been reported. Despite Ca^2+^-paradox has not been a hot topic lately, recent scientific efforts apparently still have been able to provide new insights into the mechanism of Ca^2+^-overload-induced changes.

In this study, we re-considered the myocardial contractile dysfunction due to Ca^2+^-paradox, and aimed to explore new mechanisms underlying the decreased global contractility at myofibrillar and cellular level. For this reason, beyond standard procedure of Langendorff method, we tested Ca^2+^-stimulated and Mg^2+^ ATPase activity of ventricular myofibrils as well as contractile performance of single ventricular cardiomyocytes, as yet unexamined in rat hearts exposed to Ca^2+^-paradox. Finally, we tested whether or not molecular targets such as myofilament proteins are injured.

## Materials and methods

Experimental protocols were approved by the University of Manitoba Animal Care Committee and follow the guidelines of the Canadian Council on Animal Care and the guidelines of the National Institute of Health.

### Experimental model of Ca^2+^-paradox

According to previous protocol practice [5–7], male Sprague–Dawley rat (250–300 g) hearts were isolated under ketamine and xylazine anesthesia, and were perfused with Krebs–Henseleit solution (in mM: 120.0 NaCl, 4.8 KCl, 1.2 KH_2_PO_4_, 1.25 CaCl_2_, 1.25 MgSO_4_, 25.0 NaHCO_3_, and 8.6 glucose; pH 7.4) by using Langendorff method (37 °C, 95% O_2_, 5% CO_2_). Hearts were electrically stimulated (300 beats/min) using a Phipps and Bird stimulator (Richmond, VA, USA). Water-filled latex balloon was placed into the left ventricular cavity, and connected to a transducer (model 1050, from BP-Biopac System, Inc., Goleta, CA, USA). Left ventricular developed pressure (LVDP), end-diastolic pressure (LVEDP), rate of pressure development (+dP/dt) and pressure decay (−dP/dt) were registered. Initial LVEDP was set to 10 mmHg by inflation of the balloon. Following a 20-min stabilization, hearts were randomized into three groups (*n* = 7–9/groups) and exposed either to normal Krebs–Henseleit medium containing 1.25 mM Ca^2+^ for 15 min (Control), or to Ca^2+^-depletion for 5 min (Ca^2+^free), or to Ca^2+^-repletion with normal Krebs–Henseleit solution for 10 min after 5 min of Ca^2+^-depletion (Ca^2+^-paradox). At the end of the perfusion protocols, hearts were snap frozen in liquid nitrogen and stored at −70 °C for further use.

### Myofibrillar ATPase activity measurements

LV myofibrils were isolated (*n* = 7–9/groups) as previously described [20] and were suspended in a suspension medium (in mM: 100.0 KCl, 20.0 Tris_HCl; pH 7.0). Based on earlier protocol [21], total ATPase activity was measured in the following buffer (in mM): 20.0 imidazole, 3.0 MgCl_2_, 2.0 Na_2_ATP, 5.0 NaN_3_, 50.0 KCl, 0.01 free Ca^2+^; pH 7.0. Mg^2+^ ATPase activity was determined in the same buffer, except that free Ca^2+^ was replaced by 1.0 mM EGTA. Reactions were run for 5 min at 37 °C, and then terminated by adding ice-cold 12% trichloroacetic acid. Following centrifugation, phosphate was determined in the supernatant by colorimetric method [22]. Ca^2+^-stimulated ATPase activity was calculated from the total and basal Mg^2+^ ATPase activity.

### Contractile force measurements of isolated cardiomyocytes

Contractile function of skinned ventricular cardiomyocytes (*n* = 12–13/groups) from isolated hearts perfused by Langendorff method was measured as described previously [23]. Briefly, deep-frozen (−70 °C) tissue samples were mechanically disrupted and membrane-permeabilized by 0.5% Triton X-100 detergent in isolating solution (in mM: 1.0 MgCl_2_, 100.0 KCl, 2.0 EGTA, 4.0 ATP, 10.0 imidazole; pH 7.0) at 4 °C. Each subjected cell was attached at each end to a stainless steel insect needle connecting to either a high-speed length controller (Aurora Scientific, Inc., Aurora, Canada) or a sensitive force transducer (SensoNor AS, Horten, Norway) at 15 °C. Subsequent cardiomyocyte isometric force generation was recorded at sarcomere length of 2.3 μm and analyzed by LabVIEW software (National Instruments, Corp., Austin, TX, USA). Ca^2+^-dependent force production of a single cardiomyocyte was induced by transferring the preparation from relaxing (in mM: 10.0 BES, 37.11 KCl, 6.41 MgCl_2_, 7.0 EGTA, 6.94 ATP, 15.0 creatine-phosphate; pH 7.2) to activating solution (same composition as relaxing solution aside from containing CaEGTA instead of EGTA). In our experiments, Ca^2+^ concentrations were indicated as −log_10_[Ca^2+^] units, and accordingly the pCa of relaxing solution was 9.0, whereas the pCa of maximal activating solution was 4.75. Protease inhibitors were added to all solutions freshly: phenylmethylsulfonyl fluoride (PMSF): 0.5 mM; leupeptin: 40 μM; and E-64: 10 μM. All chemicals were purchased from Sigma-Aldrich, Corp. (St. Louis, MO, USA).

Maximal and submaximal Ca^2+^-activated force generation of isolated cardiomyocytes was registered using maximal activating solution and submaximal activating solutions with different Ca^2+^ concentrations (pCa 5.4–7.0), respectively. During Ca^2+^-contractions, a so-called release–restretch maneuver, i.e., slack test, was applied in order to estimate the rate constant of force redevelopment (*k*_tr_). Actin–myosin turnover constant *k*_tr_ was determined at pCa values ranging from 4.75 to 6.0. Note that force redevelopment could not be fitted accurately at pCa > 6.0 due to the low signal-to-noise ratio. Plots indicating active force values at each pCa normalized to the corresponding maximum at pCa 4.75 as 1.0 were fitted by a specific sigmoidal function in Origin 6.0 analysis program (OriginLab, Corp., Northampton, MA, USA). It follows that the pCa value for the half-maximal contraction indicated by pCa_50_ defines *per se* the Ca^2+^-sensitivity of the contractile machinery. The steepness of the Ca^2+^-sensitivity curve, reflecting the myofilament cooperation, was calculated by a modified Hill equation and expressed as a coefficient (*n*_Hill_). Ca^2+^-independent passive force of the examined cardiac cell was measured by the shortening to 80% of initial preparation length in relaxing solution. Original forces (in µN) recorded by this experimental composition were adapted by cross section area—in µm^2^ calculating from the width and height—of each individual cell indicating cardiomyocyte tension (expressed in kN/m^2^).

### Biochemical analysis of myofilament proteins

LV myocardial tissue samples were pulverized in a chilled mortar and homogenized in an ice-cold buffer containing (in mM) 30.0 KCl, 15.0 imidazole, 5.0 NaCl, 1.0 MgCl_2_, 1.0 EGTA, 1.0 EDTA, 0.5 DTT and CaCl_2_, 0.3 Calpain Inhibitor I (Calbiochem, San Diego, CA, USA), leupeptin, and Phosphatase Inhibitor Cocktail 1 (all from Sigma-Aldrich except stated otherwise), pH 7.5. Protein concentration was determined according to the Lowry method. Homogenates were aliquoted and immediately boiled in SDS sample buffer for 3 min. To ascertain protein degradation in the crude homogenates, one-dimensional SDS-polyacrylamide gel electrophoresis (SDS-PAGE) was performed (Bio-Rad Laboratories, Inc., Hercules, CA, USA) in a non-stacking gradient gel system (concentration range: 6–18%; 30 µg protein/lane) followed by silver staining [24].

For further analysis of the samples, titin was separated by SDS-PAGE on 2% agarose-strengthened gels as detailed elsewhere [25]. Protein bands with apparent molecular mass of 3000–3300 kDa were visualized using Coomassie brilliant blue (Reanal, Ltd., Budapest, Hungary). Suspected small myofilament proteins were separated on single-concentration SDS-polyacrylamide gels (α-actinin: 7%; desmin: 10%; actin, cardiac troponin T (cTnT), tropomyosin (Tm), and cardiac troponin I (cTnI): 15%; myosin light chain-1 and -2 (MLC-1 and MLC-2, respectively): 20%; Mini Protean II, Bio-Rad; 5–10 µg protein/lane), transferred appropriately and identified by Western immunoblot (Bio-Rad). Enhanced chemiluminescent (ECL) detection was used for protein visualization as previously used [26]. Membranes were probed with the following primary antibodies: anti-*α*-actinin clone EA-53 (Sigma-Aldrich), dilution 1:5000; anti-desmin clone DE-U-10 (Sigma-Aldrich), dilution 1:7000; anti-actin (Abcam, Cambridge, UK), dilution 1:1000; anti-cTnT clone 1A11 (Research Diagnostics, Inc., Flanders, NJ, USA), dilution 1:3000; anti-Tm (Sigma-Aldrich), dilution 1:600; anti-cTnI clone 19C7 (Research Diagnostics), dilution 1:1000; anti-MLC-1 (Santa Cruz Biotechnology, Dallas, TX, USA), dilution 1:7000; and anti-MLC-2 (Abcam), dilution 1:400. Peroxidase-conjugated secondary antibody was used as appropriate (Sigma-Aldrich), dilution 1:3000.

For myosin heavy chain (MHC) isoform analysis with Coomassie blue staining, rat ventricular tissues were homogenized under denaturing conditions. MHC isoforms (α and β) were separated on a 4% polyacrylamide gel as described previously [27, 28]. 4 µg of protein sample was loaded in each well. Electrophoresis was carried out at a constant 220 V for 3–3.5 h at around 15 °C. Gels were stained with Coomassie brilliant blue R-250 (Bio-Rad) for 2 h, and then destained with 7% acetic acid by diffusion. Human failing heart ventricular sample served as α-MHC and β-MHC isoform control. MHC isoforms were documented by GS-670 Imaging Densitometer (Bio-Rad).

For MHC Western immunoblot analysis, cardiac tissues were prepared as for the mechanical measurements, except that the isolated samples were dissolved in sample buffer instead of isolating solution. Male Sprague–Dawley soleus muscle was used as β-MHC control. MHC isoforms were separated on SDS-PAGE following earlier protocol [29] with modifications. Then proteins were transferred to nitrocellulose membrane and probed with primary (pan anti-MHC, dilution 1:10000, from Sigma-Aldrich; and anti-β-MHC isoform: mouse IgM, MYH7 (A4.840) sc-53,089, dilution 1:1000, from Santa Cruz Biotechnology, Inc., Dallas, TX, USA) and secondary antibodies (peroxidase-conjugated secondary antibody, dilution 1:3000, from Sigma-Aldrich; and goat anti-mouse IgM, 115-035-075, dilution 1:20000, from Jackson ImmunoResearch Laboratories, Ltd., West Grove, PA, USA) as appropriate. Proteins were detected by ECL reaction and documented by MF-ChemiBIS 3.2 gel documentation system (DNR Bio-Imaging Systems, Ltd., Jerusalem, Israel). Signal intensities were evaluated by ImageJ 1.41o image processing program (National Institutes of Health, Bethesda, MD, USA). All biochemical analyses were performed on 4–6 samples/groups.

### Statistical analysis

Data are expressed as mean ± SEM. Statistical analysis was performed with GraphPad Prism 5.02 software (GraphPad Software, Inc., La Jolla, CA, USA). Differences were evaluated by one-way ANOVA followed by Bonferroni post hoc test. *P* values of <0.05 were considered statistically significant.

## Results

### Ca^2+^-paradox dramatically depresses LV contractile function in Langendorff hearts

**Fig. 1:**
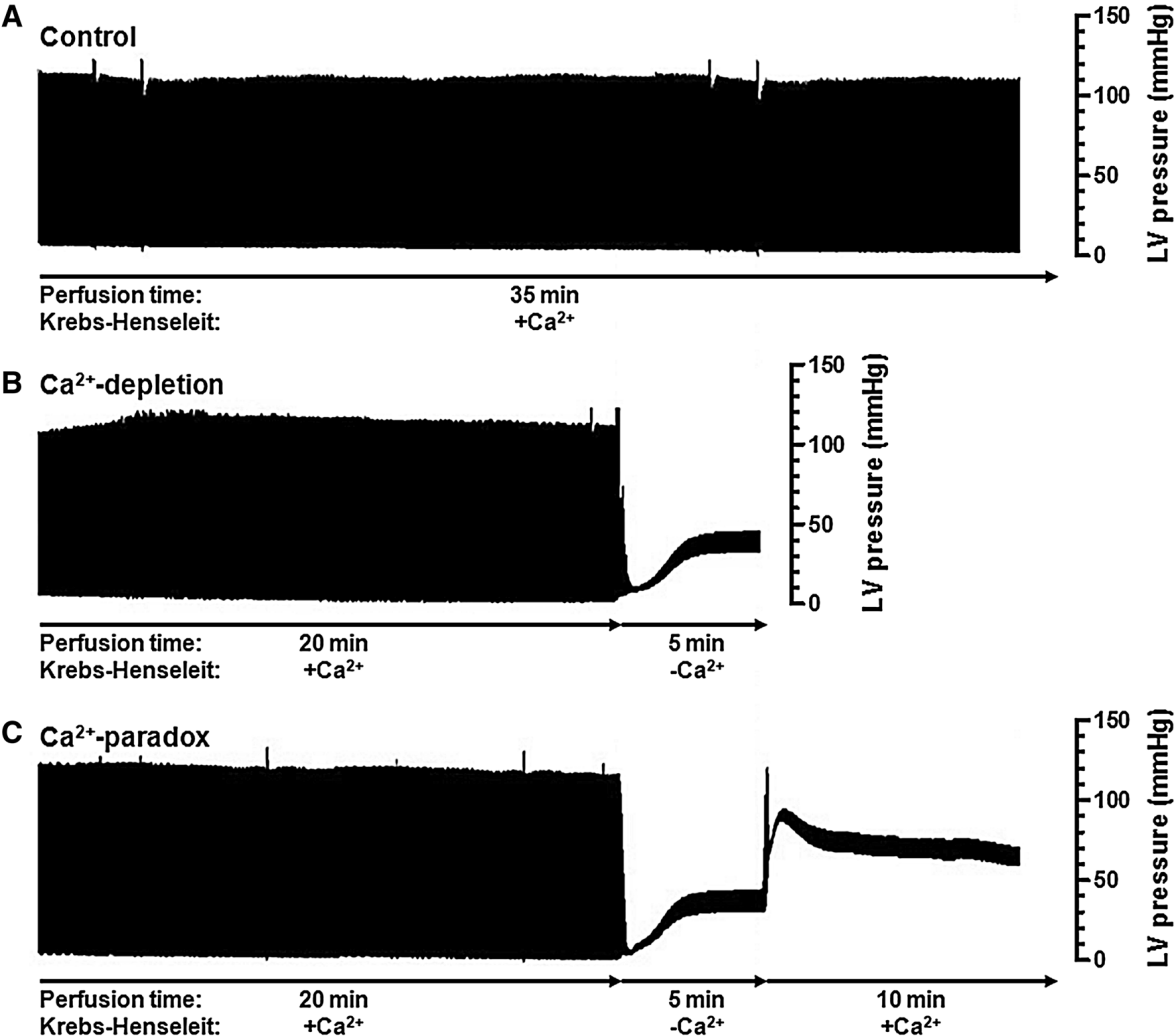
Experimental model design and representative records of LV pressure measurements are demonstrated by Langendorff method. LV pressure is shown as a function of time in Control rat hearts (**a**), Ca^2+^-depleted rat hearts (**b**), and rat hearts exposed to Ca^2+^-paradox (**c**)

LVDP recorded in Control hearts (127.4 ± 6.1 mmHg) was permanently decreased during both Ca^2+^-depletion (9.8 ± 1.3 mmHg, *P* < 0.001) and Ca^2+^-paradox (12.9 ± 1.3 mmHg, *P* < 0.001; Fig. 1; Table 1). Similarly, +dP/dt and –dP/dt in Ca^2+^-depletion (62.6 ± 6.9 and −87.9 ± 12.6 mmHg/sec, respectively, *P* < 0.001), as well as in Ca^2+^-paradox (93.9 ± 19.7 and −163.4 ± 17.9 mmHg/sec, respectively, *P* < 0.001) were significantly lower than in Controls (7073.8 ± 351.0 and −4075.3 ± 206.2 mmHg/sec, respectively; Fig. 1; Table 1). At the same time, LVEDP of Control hearts (4.1 ± 0.5 mmHg) was markedly increased due to both Ca^2+^-depletion (32.2 ± 3.3 mmHg, *P* < 0.001) and Ca^2+^-paradox (72.4 ± 5.0 mmHg, *P* < 0.001; Fig. 1; Table 1).

**Table 1:** LV contractile parameters in Control, Ca^2+^-depleted, and Ca^2+^-paradox rat hearts are given by Langendorff method

*Group*	Control	Ca^2+^-depletion	Ca^2+^-paradox
		(*n *= 7–9)	
*Hemodynamic parameters*			
LVDP, mmHg	127.4 ± 6.1	9.8 ± 1.3*	12.9 ± 1.3*
LVEDP, mmHg	4.1 ± 0.5	32.2 ± 3.3*	72.4 ± 5.0*^,#^
+dP/dt, mmHg/sec	7073.8 ± 351.0	62.6 ± 6.9*	93.9 ± 19.7*
−dP/dt, mmHg/sec	−4075.3 ± 206.2	−87.9 ± 12.6*	−163.4 ± 17.9*

Data are given as mean ± SEM

*LVDP* LV developed pressure, *LVEDP* LV end-diastolic pressure, *+dP/dt* rate of pressure development in the LV, *−dP/dt* rate of pressure decay in the LV

*P < 0.05 versus Control; ^#^ *P* < 0.05 versus Ca^2+^-depletion

### Myofibrillar Ca^2+^-stimulated ATPase activity is decreased, while Mg^2+^ ATPase activity is increased in Ca^2+^-paradox

Compared to Control hearts (12.08 ± 0.57 µmol P_i_/mg protein/h), myofibrillar Ca^2+^-stimulated ATPase activity was decreased upon Ca^2+^-depletion (8.13 ± 0.19 µmol P_i_/mg protein/h, *P* < 0.001) as shown in Table 2, and it remained low in Ca^2+^-paradox (8.40 ± 0.22 µmol P_i_/mg protein/h, *P* < 0.001; Table 2). In contrast, Control Mg^2+^ ATPase activity (3.20 ± 0.25 µmol P_i_/mg protein/h) was not altered by Ca^2+^-depletion (3.27 ± 0.10 µmol P_i_/mg protein/h), but it was increased through Ca^2+^-paradox (7.21 ± 0.36 µmol P_i_/mg protein/h, *P* < 0.001; Table 2).

**Table 2:** Basal Mg^2+^-dependent and Ca^2+^-stimulated myofibrillar ATPase activities are shown in Control, Ca^2+^-depleted, and Ca^2+^-paradox ventricles

*Group*	Control	Ca^2+^-depletion	Ca^2+^-paradox
		(*n* = 7–9)	
*ATPase activity*			
Basal (Mg^2+^), µmol P_i_/mg protein/h	3.20 ± 0.25	3.27 ± 0.10	7.21 ± 0.36*^,#^
Ca^2+^-stimulated, µmol P_i_/mg protein/h	12.08 ± 0.57	8.13 ± 0.19*	8.40 ± 0.22*

Data are given as mean ± SEM

*P < 0.05 versus Control; ^#^ *P* < 0.05 versus Ca^2+^-depletion

### Ca^2+^-paradox impairs Ca^2+^-activated force generation of isolated cardiomyocytes

**Fig. 2:**
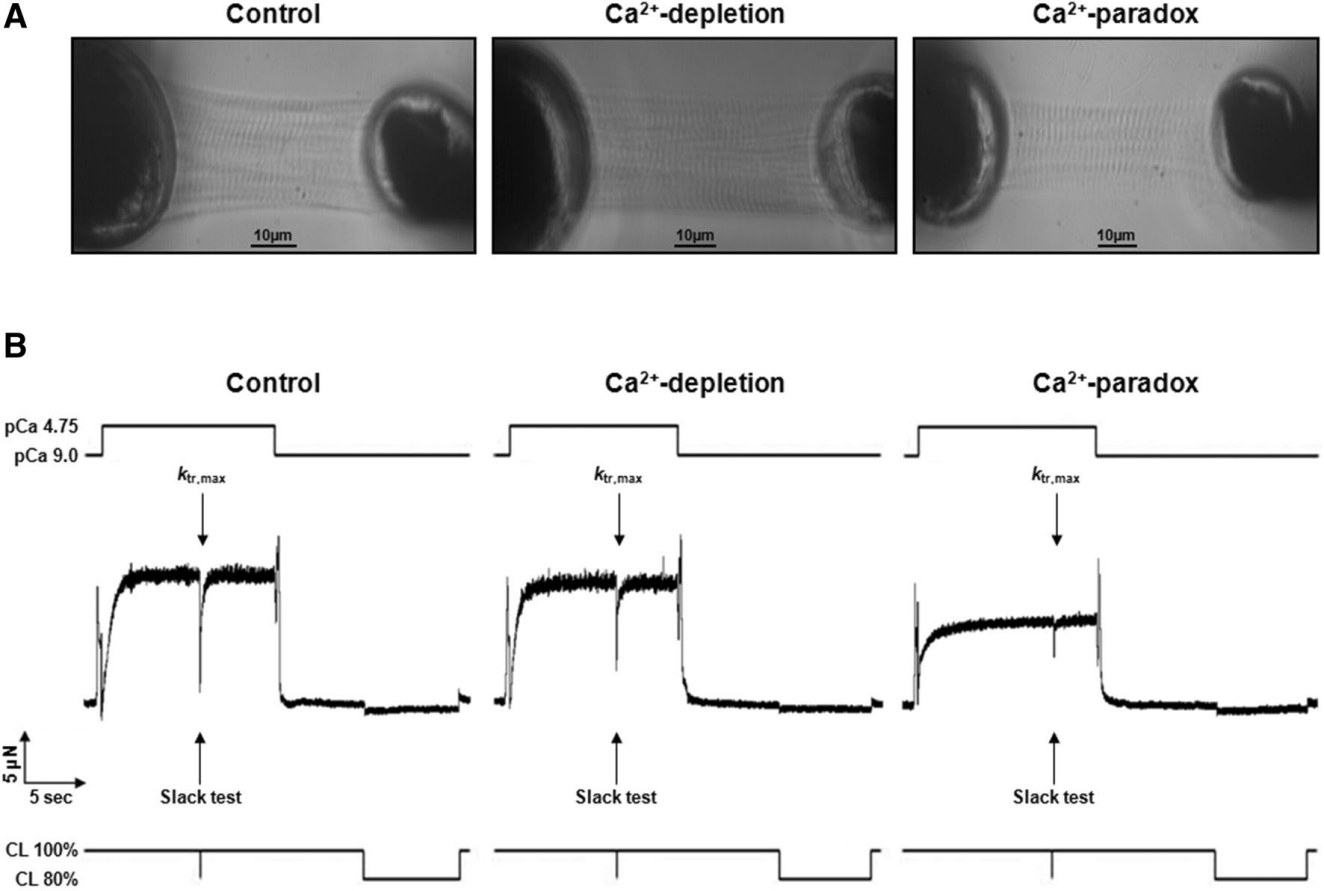
Typical appearance and function of the contractile machinery are shown in Control, Ca^2+^-depletion, and Ca^2+^-paradox groups. **a** Each demembranated myocyte-sized preparation was mounted with silicone adhesive between a high-speed length controller (*left*) and a sensitive force transducer (*right*). Sarcomere length was set to 2.3 µm. *Scale bars* represent 10 µm. Note that apparently functional cardiomyocytes with plain cross striation pattern were selected and investigated. **b** Corresponding original force recordings demonstrate experimental design of cellular force measurements. Maximal Ca^2+^-activated force generation was induced by transferring the preparation from relaxing solution with −log_10_[Ca^2+^] (pCa) 9.0 to maximal activating solution with pCa 4.75. Rapid slack test was performed at pCa 4.75 for the determination of maximal rate constant of force regeneration (*k*_tr,max_). In relaxing solution again long slack test was then performed to measure Ca^2+^-independent force. 80% of initial cell length (CL) was considered as slack length

**Fig. 3:**
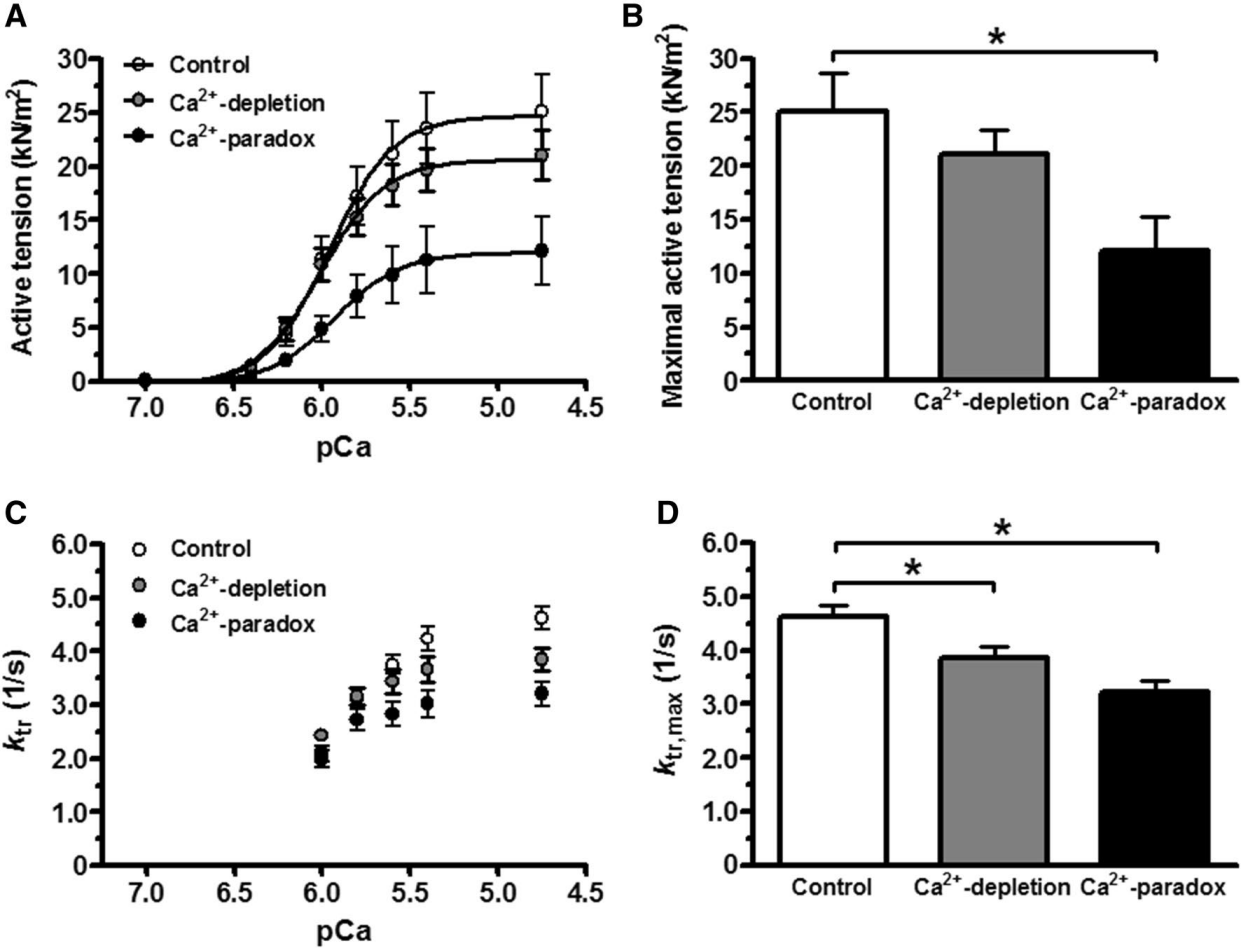
Contractile performance of isolated cardiomyocytes is shown in Control, Ca^2+^-depleted and Ca^2+^-paradox groups. **a** Cardiomyocyte active tension is shown as a function of pCa. Maximum points of active tension versus pCa relationships (at pCa 4.75) are compared in **b**. **b** Maximal active tension values of the subjected cardiomyocytes are compared on* bar graphs*. **c** Rate constant of force redevelopment (*k*_tr_) of the contractile machinery is shown as a function of pCa. **d** Maximal *k*_tr_ values at saturating Ca^2+^ levels (*k*_tr,max_) are compared on* bar graphs*. Data are given as mean ± SEM; *n* = 12–13 cells/group; *P < 0.05 versus Control

Isolated and mounted cardiomyocytes from the Control, Ca^2+^-depletion, and Ca^2+^-paradox groups all had plain cross striation pattern (Fig. 2a). Corresponding original force recordings are shown in Fig. 2b. Either active tension (Fig. 3a) or *k*_tr_ (Fig. 3c) versus pCa relationships of isolated cardiomyocytes presented obvious differences in Ca^2+^-related force production between the experimental groups. Maximal active tension of the Ca^2+^-depletion group was not significantly different (21.04 ± 2.32 kN/m^2^) from the Control values (25.07 ± 3.51 kN/m^2^), but in the Ca^2+^-paradox group it was significantly lower (12.12 ± 3.19 kN/m^2^, *P* < 0.05; Fig. 3b). Relative to Control *k*_tr_ at saturating Ca^2+^ levels (*k*_tr,max_, 4.61 ± 0.22), myocytes from the Ca^2+^-depleted hearts had lower *k*_tr,max_ (3.85 ± 0.21, *P* < 0.05), such as those from Ca^2+^-paradoxical hearts (3.21 ± 0.23, *P* < 0.001; Fig. 3d).

**Fig. 4:**
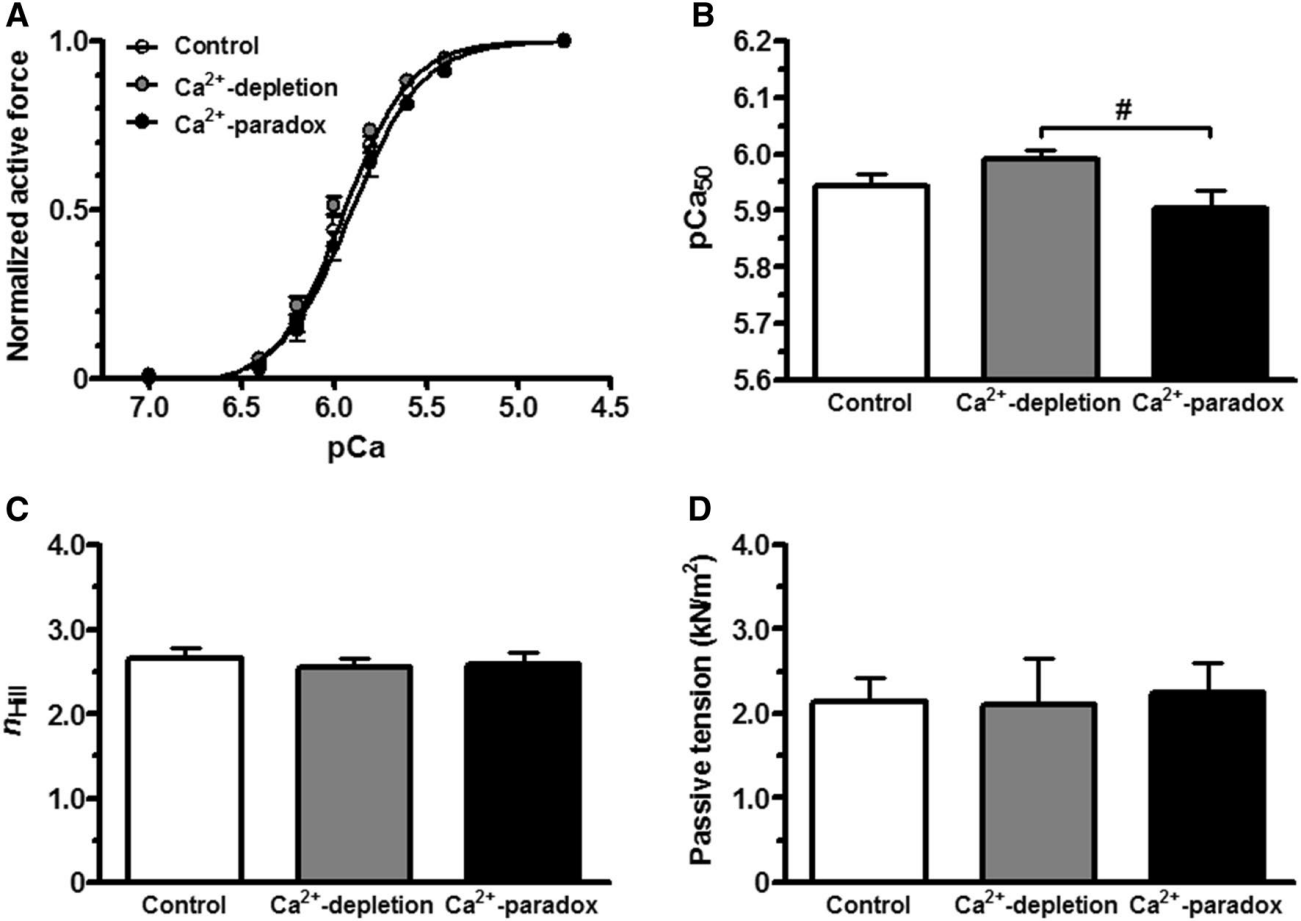
Ca^2+^-sensitivity, myofilament cooperation, and passive tension of cardiomyocytes are shown in Control, Ca^2+^-depleted, and Ca^2+^-paradox groups. **a** Normalized active force–pCa relationships provide the sigmoidal Ca^2+^-sensitivity curves of myocyte subjects. **b** Ca^2+^-sensitivity of the contractile apparatus (pCa_50_) is shown on *bar graphs*. **c** Steepness of the normalized active force versus pCa curves represented by the Hill coefficient (*n*_Hill_) demonstrates myofilament cooperation in isolated cardiomyocytes. **d** Ca^2+^-independent passive tension of the same cells is shown at pCa 9.0. Data are given as mean ± SEM; n = 12–13 cells/group; ^#^ *P* < 0.05 versus Ca^2+^-depletion

Regarding Ca^2+^-sensitivity of the subjected cardiomyocytes (Fig. 4a), pCa_50_ value was 5.99 ± 0.02 in the Ca^2+^-depletion group, which was not significantly different from the pCa_50_ of the Controls (5.94 ± 0.02; Fig. 4b). However, pCa_50_ was 5.90 ± 0.03 after Ca^2+^-repletion, indicating lower Ca^2+^-sensitivity than that in Ca^2+^-depletion (*P* < 0.05; Fig. 4b). The *n*_Hill_ values were not different between the Control, Ca^2+^-depletion, and Ca^2+^-paradox groups (2.65 ± 0.12, 2.56 ± 0.10, and 2.59 ± 0.14, respectively; Fig. 4c). Figure 4d shows cardiomyocyte Ca^2+^-independent passive tension. Relative to Control (2.13 ± 0.29 kN/m^2^), no change was observed in passive tension neither upon Ca^2+^-depletion (2.11 ± 0.54 kN/m^2^) nor upon Ca^2+^-paradox (2.25 ± 0.35 kN/m^2^; Fig. 4d).

### Ca^2+^-paradox disturbs myofibrillar integrity, including α-MHC and cTnT

**Fig. 5:**
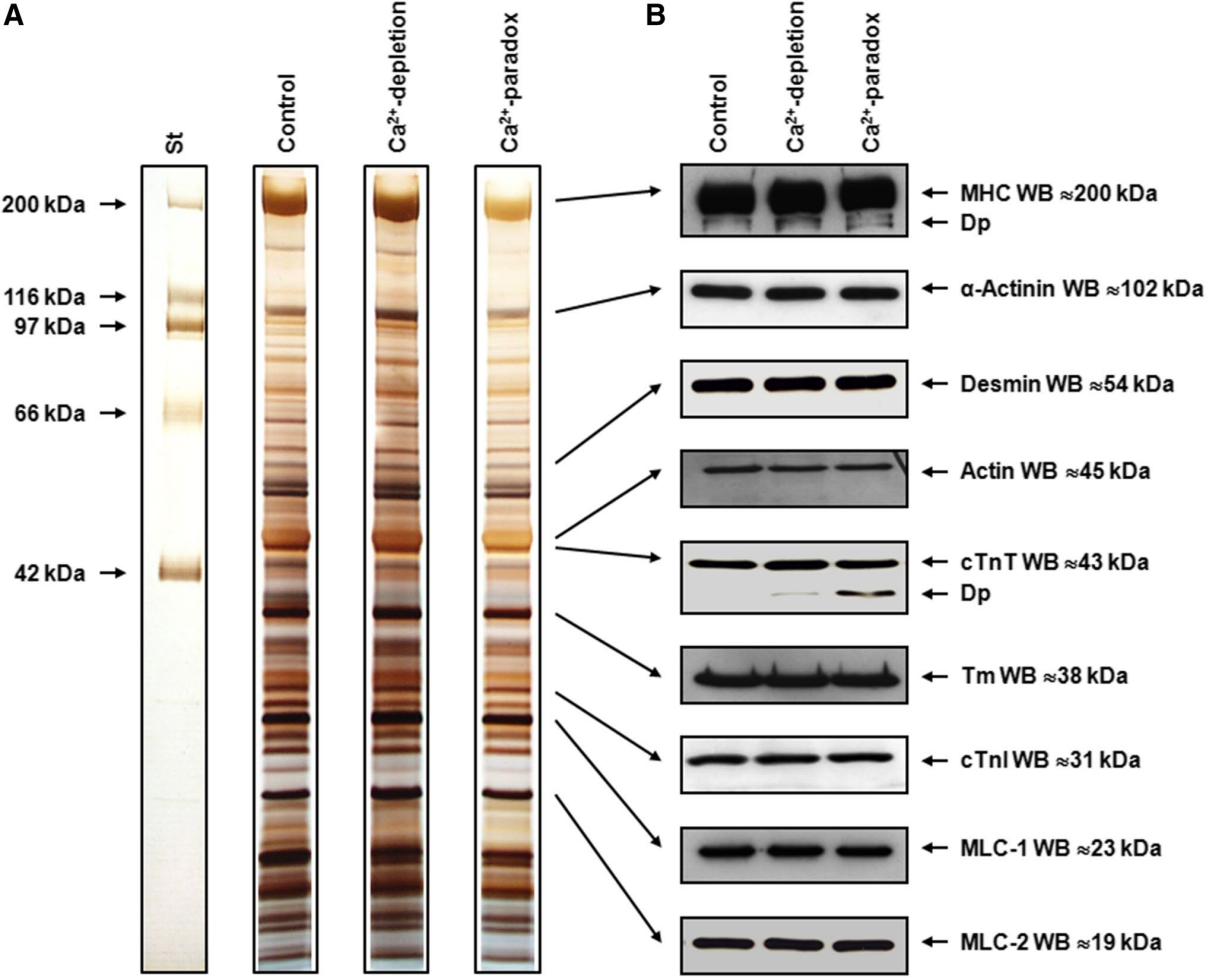
Ventricular homogenates from Langendorff-perfused rat hearts were tested for protein degradation. **a** Representative protein compositions (≤200 kDa) are shown on 6–18% gradient polyacrylamide gels by silver staining. One lane is shown of molecular weight standards (St), and Control, Ca^2+^-depletion, and Ca^2+^-paradox samples, respectively. Samples were run together, where* black frames* indicate gel discontinuity. **b** Myofilament proteins were identified and tested for degradation by Western immunoblot (WB). Corresponding images (*uncut between lanes*) are representative examples. Protein bands around 200, 102, 54, 43, 38, 31, 23, and 19 kDa were co-migrated with myosin heavy chain (MHC), α-actinin, desmin, actin, cardiac troponin T (cTnT), tropomyosin (Tm), cardiac troponin I (cTnI), myosin light chain-1 (MLC-1), and myosin light chain-2 (MLC-2), respectively

**Fig. 6:**
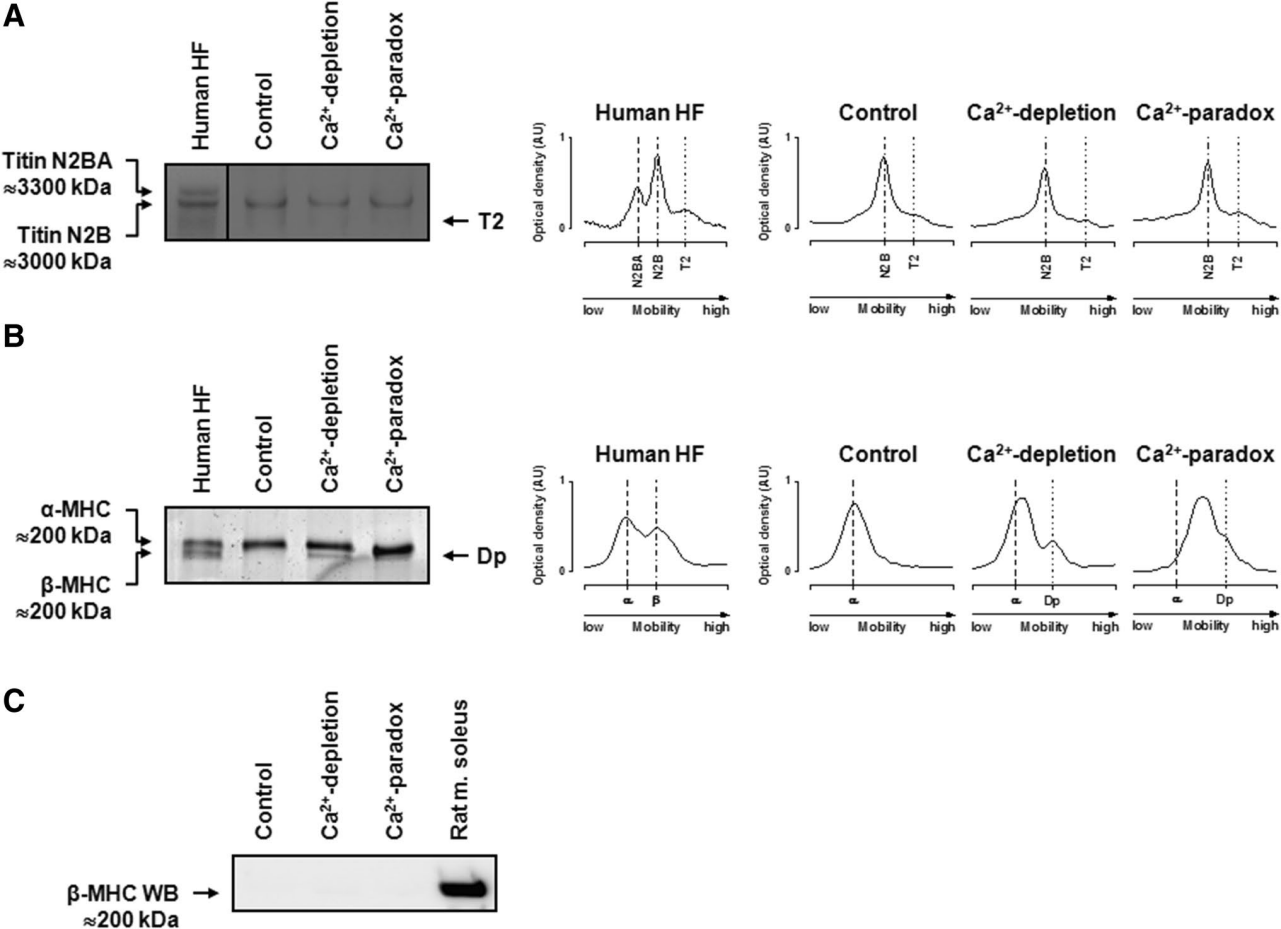
Titin and myosin heavy chain (MHC) isoform compositions are shown in Control, Ca^2+^-depletion, and Ca^2+^-paradox ventricular samples. **a** Representative image shows titin isoform separation on agarose-strengthened polyacrylamide gels. Protein bands were visualized with Coomassie. *Black line* indicates discontinuity between *lanes*. Human heart failure (HF) ventricular sample served as titin isoform control showing protein bands with lower mobility around 3300 kDa (N2BA) and with higher mobility around 3000 kDa (N2B). T2 band is considered as a barely detectable large titin degradation product. Corresponding optical densities—in arbitrary units (AU)—show only N2B titin isoform with similar mobility and no apparent degradation in Control, Ca^2+^-depletion, and Ca^2+^-paradox groups. Three titin separations in duplicates in all experimental groups gave similar results. **b** MHC isoforms were separated on polyacrylamide gels and stained with Coomassie. Human HF ventricular sample served as α-MHC and β-MHC isoform control where both isoforms could be detected. Only one MHC isoform could be identified in Control samples at the level of the α-MHC isoform. Appearance of the lower band(s) in the Ca^2+^-depletion and Ca^2+^-paradox groups supports protein degradation. Corresponding optical densities—in AU—confirm higher mobility of the parent MHC molecule (α isoform) and the co-appearance of a degradation product (Dp) in Ca^2+^-depletion and Ca^2+^-paradox groups. Five different MHC separations in all experimental groups gave similar results. **c** The apparent lack of protein bands around 200 kDa co-migrating with β-MHC isoform in Control, Ca^2+^-depletion, and Ca^2+^-paradox groups is demonstrated by Western immunoblot (WB). Rat *musculus* (m.) soleus skeletal muscle was used as antibody control

The protein composition of myocardial homogenates (≤200 kDa) is shown with silver staining in Fig. 5a. In order to identify potential degrading proteins, we have tested the integrity of several myofibrillar proteins using Western immunoblot (Fig. 5b). Pan MHC Western immunoblot clearly identified additional bands with higher mobility than the parent molecules in Ca^2+^-depletion and Ca^2+^-paradox groups (Fig. 5b), suggesting MHC degradation. As explained by the species-dependent MHC isoform expression, β-MHC isoform was not recognized by Western immunoblot in our rat hearts (Fig. 6c). Therefore, the MHC we see in Fig. 5b is α-MHC, since only that isoform was expressed in our samples. The specific electrophoretic separation of MHC isoforms followed by Coomassie staining also confirms that the control samples express only the α-MHC isoform (Fig. 6b) and this latter isoform showed up as a somewhat lower band in the Ca^2+^-depletion and Ca^2+^-paradox groups compared to the parent molecule of the Controls, indicating protein degradation (Fig. 6b). Moreover, cTnT also exhibited a progressive degradation during Ca^2+^-depletion then Ca^2+^-repletion as seen in Fig. 5b. In contrast, we found no changes in the protein amount and integrity of α-actinin, desmin, actin, Tm, cTnI, MLC-1, and MLC-2 between the experimental groups (Fig. 5b). Titin did not show apparent degradation in Ca^2+^-depletion and Ca^2+^-paradox (Fig. 6a).

## Discussion

For the first time, this study aimed to investigate the influence of Ca^2+^-paradox on cardiac function at cellular and myofibrillar level. Here we demonstrate that global contractile impairment of isolated hearts due to Ca^2+^-depletion then Ca^2+^-repletion is associated with altered myofibrillar ATPase activity and decreased Ca^2+^-dependent force production of single skinned cardiomyocytes. Furthermore, degradation of contractile proteins—such as α-MHC and cTnT—occurs in hearts affected by Ca^2+^-paradox. These data suggest an insufficient actin–myosin interaction in intracellular Ca^2+^-overload, leading to contractile dysfunction presumably on the basis of myofibrillar damage.

In accordance with prior findings in Ca^2+^-paradox [5, 6], we observed global LV systolic dysfunction in both Ca^2+^-depletion and Ca^2+^-paradox indicated by a low LVDP associated with a uniform fall in +dP/dt and −dP/dt. At the same time, as a result of the significant rise in LVEDP during Ca^2+^-depletion then Ca^2+^-replacement, these hearts manifested diastolic dysfunction as well. This observation is in line with earlier studies reporting increased resting tension in Ca^2+^-paradox [17]. Hypercontracture in Ca^2+^-paradoxical hearts [4, 8, 10] is therefore likely to be functionally considered as a pathological condition of inadequate contraction and relaxation side by side.

Chemical energy required for myofilament contraction originates from ATP hydrolysis through myosin ATPase, while ATP binding again to myosin heads is needed for cardiac muscle relaxation cycle-to-cycle [30, 31]. Basal ATP hydrolysis by the Mg^2+^-dependent myosin ATPase activity triggers myosin off to a high-energy form binding P_i_ and ADP. Ca^2+^-induced conformational changes in the troponin complex relieve myofilaments of inhibition making actin accessible for myosin heads, thereby facilitating cross-bridge formation. Dissociation of the P_i_ from the bounded myofilaments is mediated by the Ca^2+^-stimulated myosin ATPase activity, initiating the power stroke. The subsequent release of ADP leads to rigor, in other words nucleotide-free myosin in low-energy form. The new coupling of ATP and myosin heads finally results in relaxation, detaching myosin from actin.

An increased diastolic tension and pressure therefore raise the lack of ATP as required for relaxation. Cytosolic ATP content, however, is not only preserved during hypercontracture that is provoked by intracellular Ca^2+^-overload, but ATP has been demonstrated as a prerequisite in the development of Ca^2+^-paradox [32]. Nevertheless, studies of rigor tension on isolated skinned rat cardiomyocytes have revealed the possibility of ATP compartmentalization in ischemic contracture [33]. Accordingly, under certain pathological conditions [Mg^2+^-ATP] might differ in the myofibrils and in the cytosol. Based on this theory, one could speculate that depletion of ATP in the myofilament compartment might lead to impaired relaxation [34], thereby hypercontracture in Ca^2+^-paradoxical hearts, even if cytosolic ATP supply is preserved. Nonetheless, it has been already reported that contracture development is promoted by a rise in intracellular [Ca^2+^] by activating ATPases, and consequently internal Ca^2+^-overload promotes ATP depletion and accelerates rigor tension development [3]. In myofibrils from isolated hearts suffered Ca^2+^-paradox, we documented an enhanced Mg^2+^-dependent ATPase, but a depressed Ca^2+^-stimulated ATPase activity. This observation implies that in Ca^2+^-paradox a higher rate of basal ATP hydrolysis on myosin heads is followed by a lower dissociation rate of ATP metabolites, contributing to stiffer actin–myosin interaction and/or local ATP consumption under relaxed conditions. In the regulation of cardiac contraction, it is generally accepted that Mg^2+^-ADP binds strongly to myosin, promoting the isometric tension and decreasing tension kinetics [23]. Experiments on rat skinned fibers have demonstrated that Mg^2+^-ADP has a clear impact on rigor tension development as well as on myosin ATPase activity, suggesting that development of rigor cross-bridges might be related to an increase in myosin ATPase activity [34]. Indeed, in rat permeabilized cardiomyocytes rigor was associated with enhanced myosin ATPase activity; in addition, Mg^2+^-ADP was shown to stimulate myosin ATPase [35]. These results propose an inhibitory effect of Mg^2+^-ADP on dissociation steps and/or on further Mg^2+^-ATP binding and cross-bridge detachment [34]. Since maximal speed of contraction is presumably controlled by the rate of cross-bridge detachment [30], the release of Mg^2+^-ADP becomes the main rate-limiting step in cross-bridge cycling, thereby in contracture development [34, 35]. Accordingly, it appears that the stimulated myosin ATPase in Ca^2+^-paradox is the Mg^2+^-dependent one, resulting in higher rate of ATP hydrolysis along with slower rate of P_i_ and ADP release. This apparent discrepancy might lead to a proportionally increased number of myosin heads bounded to actin in each cross-bridge cycle. The stiff actin–myosin interaction and/or elevated basal ATP consumption then might result in high resting tension. This theory might explain increased LVEDP seen in Ca^2+^-paradox, despite the fact that diastolic intracellular [Ca^2+^] is relatively low during Ca^2+^-paradox [36]. On the other hand, we found that in Ca^2+^-depleted and Ca^2+^-paradoxical hearts, global impairment of systolic function was associated with depressed myofibrillar Ca^2+^-stimulated ATPase activity. It has been known for a long time that myosin ATPase determines the velocity of muscle contraction [37], and thus indices of Ca^2+^-activated contraction rate (e.g., dP/dt) are tightly correlated with Ca^2+^-stimulated ATPase activity of myofibrils as elementary for initiating power stroke [30]. Consequently, low Ca^2+^-stimulated ATPase activity is associated with reduced Ca^2+^-dependent contraction, resulting in systolic dysfunction, like in Ca^2+^-paradox hearts.

Beyond that, for the first time we made an attempt to test cellular mechanical function in Ca^2+^-paradox, and described a significant decrease in maximal active tension and *k*_tr,max_ of still surviving isolated cardiomyocytes after Langendorff-perfusion. These results from single-cell preparations are consistent with our LV contractility findings, since we consider reduced active tension as the cellular basis of depressed LVDP. Similar drop in +dP/dt and −dP/dt is the global reflection of reduced *k*_tr,max_ by reason of depressed Ca^2+^-stimulated ATPase activity. In contrast to Ca^2+^-dependent parameters, passive tension of isolated cardiomyocytes was not affected at all during Ca^2+^-depletion then Ca^2+^-replacement. Thus, passive myofilament components of diastole appear unlikely to be primarily responsible for myocardial stiffening in Ca^2+^-paradox.

Here we detected a gradual damage of essential contractile proteins, such as α-MHC and cTnT en route to Ca^2+^-paradox. Based on previous observations, limited degradation of myofibrils might occur after Ca^2+^-paradox, suggesting cTnT release through membrane destruction [38]. Likewise homogenates from guinea pig hearts exposed to Ca^2+^-overload exhibited degradation of cTnT. A potential cross-linking between troponin subunits or their fragments and other cardiac proteins was therefore suggested in cell death after Ca^2+^-paradox [39]. These results are clearly in accordance with our findings based on Western blot assays suggesting progressive cTnT degradation upon Ca^2+^-depletion and Ca^2+^-repletion, although we did not observe cross-linked cTnT forms.

In summary, isolated rat hearts suffered Ca^2+^-paradox demonstrate impaired global and cellular contractility that is accompanied by decreased Ca^2+^-stimulated ATPase activity potentially as a result of myofilament protein degradation. According to data provided in this study, we conclude that significant deterioration in cardiac relaxation seen in Ca^2+^-paradoxical hearts is probably due to a failure in cross-bridge cycling because of an altered myofibrillar ATPase activity, and is apparently not directly related to titin. On the other hand, it is likely that one of the molecular bases of dramatic decrease in systolic function in Ca^2+^-paradox might be α-MHC and cTnT cleavage, resulting in a collective reduction of interdependent parameters of Ca^2+^-dependent force generation at the level of cardiomyocytes, myofibrils, and isolated heart.
